# In Vision It Is Groups, Rather Than Maps, That Determine How We Perceive the World

**DOI:** 10.3390/vision6030051

**Published:** 2022-08-19

**Authors:** Philip T. Quinlan, Keith Allen, Dale J. Cohen

**Affiliations:** 1 Department of Psychology, The University of York, Heslington, York YO10 5DD, UK; 2 Department of Philosophy, The University of York, Heslington, York YO10 5DD, UK; 3 Department of Psychology, The University of North Carolina at Wilmington, Wilmington, NC 28403, USA

**Keywords:** Boolean map theory, perceptual grouping, human visual processing, object counting

## Abstract

This paper presents the results of a study that used a speeded counting task to adjudicate between two competing theories of how perceptual representations of visual objects are derived. Boolean map (BM) theory assumes that there are strict limits on conscious awareness, such that we only have serial access to features on the same dimension (e.g., red and green). This theory contrasts with views that emphasize the early grouping of features, and which assume that feature processing is interactive and underpins figure/ground segregation as a necessary precursor to object perception. To test between these theories, we report performance in a speeded counting task in which participants were asked to judge which of two shapes was more prevalent. Displays contained squares and circles that appeared in either of two colors, with color and shape distinctions either perfectly correlated (i.e., compatible) or not (i.e., incompatible). BM theory predicts no influence of the relative coincidence of color and shape on the identification of the more prevalent shape. In contrast, grouping theory predicts that performance will be better when the color/shape distinction is compatible than when it is incompatible. Our data strongly support the grouping theory predictions. We conclude that the primary constraints on how visual objects are accessed are the number and kind of groupings that are recovered, not the number of feature maps consulted.

## 1. Introduction

How are perceptual representations of visual objects derived? It is commonly assumed that, at a very early stage of visual processing, perceptible features (e.g., color, shape, and motion) are registered on separate, functionally independent, feature maps [1]. In setting out Boolean map (BM) theory, Huang and colleagues [2,3,4,5,6] adopted these architectural assumptions and used them to make claims about functional constraints that operate when people see. One particularly striking claim is that the visual system undertakes a “divide and conquer” approach, with the constituent features of the visual world being registered on corresponding feature maps, and information from these maps then being used to construct subsequent representations of the optic array in terms of what are known as ‘Boolean maps’. The aim of this study is to assess the BM theory of how perceptual representations of visual objects are derived, and compare it with a competing account that instead emphasizes the early grouping of features.

In general terms, a given Boolean map codes the locations of items that share a common featural value. For instance, all red items would be captured on a RED Boolean map. There is not one ‘color map’ per se, but different maps for particular colors: for example, one map for red items and a different map for green. Once such a map has been constructed for a particular featural value, all the other properties of items coded on the map can then be accessed. Thus, once a red color map has been constructed, squares that are red can be identified. Critically though, only one such map can be processed at a time. As a consequence, red and green items cannot be processed in parallel, and neither can red items and squares.

Given this strict serial constraint, very particular predictions have been derived. For example, the theory predicts that the time to judge the symmetry of a matrix of colored square patches will scale as a function of the number of types of colors that are present, and, indeed, this is exactly what has been found [7,8].

Since its inception, the theory has developed and much evidence has been marshalled in its support [2,3,4]. We provide detailed discussion of some key aspects of this work as the material unfolds. We begin, however, with a recent development in which contrasts have been drawn between predictions derived from BM theory and those derived from theories that give a prominent role to grouping. Huang [4] reported findings from an experiment on visual short-term memory in which the to-be-remembered display contained six color discs. In some displays the disks were spatially separate, and in others, pairs of discs were configured as dumbbells—discs connected by a single bar. According to one grouping hypothesis, memory ought to be facilitated for the dumbbell configurations relative to the spatially separate discs. This is based on the assumption that fewer object-based representations are encoded and stored in short-term memory in the dumbbell case than the disc case (i.e., three vs. six objects, respectively). However, in probing memory for the color of one of the discs selected at random, Huang failed to show any additional memory benefit when the discs were connected versus when they were not. This evidence was taken as being more in line with featural-based processing, and hence BM theory, rather than object-based processing based on grouping by connectedness.

As with any null effects, interpretation is difficult and partly because of this we were motivated to compare and contrast BM theory with a theory of processing based on more general Gestalt principles of grouping that go beyond connectedness. Fundamentally, the ideas about Gestalt grouping are that plausible figures are separated from their background by interactive activation and the competitive processing of the current perceptual features; that is, figure/ground segregation is facilitated when information across different perceptual dimensions suggests the same parcellation of the input, and inhibited when cues on different dimensions suggest different groupings [9,10,11]. The central idea is that basic perceptual processes operate from the bottom–up in such a way that plausible best guesses are made about possible objects in the immediate environment. Further processing can then be directed towards object-based representations.

This grouping account contrasts with BM theory in this respect, which simply assumes that the only significant processing constraints operate once object-based representations have been derived. The theory posits that figure/ground segregation is a necessary step prior to accessing an object’s properties. Such property access is then constrained by the construction and consultation of information specified on at least one Boolean map. There is no notion of facilitation across different perceptual dimensions. There are only time costs associated with the construction and recovery of featural information from independent Boolean maps.

**Figure 1:**
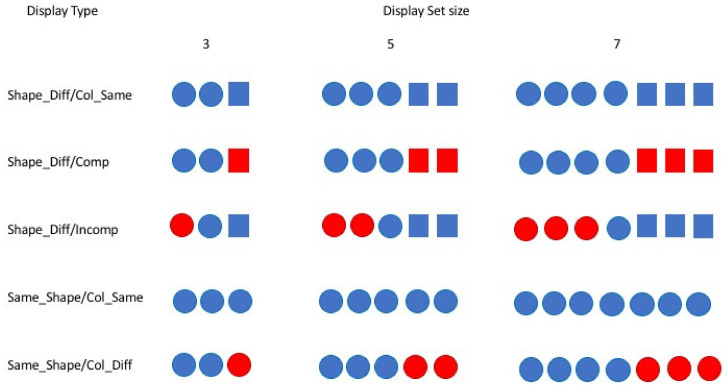
Examples of the composition of the displays used. The figure does not reflect the spatial layout of the shapes in the displays. The individual shapes were positioned randomly within a virtual rectangle centered at fixation and the positions of the shapes were determined at random prior to each trial. An important constraint was that none of the shapes could overlap.

Given the contrasting nature of the two theories, we aimed to derive different sets of predictions of performance in a novel speeded shape-counting task, pitting the featural processing account of BM theory in which the number of feature maps is critical against a grouping account in which it is the number of featural groups that is critical. In this task, participants were presented with visual displays containing colored squares and circles and were instructed to judge whether squares or circles were more prevalent and respond accordingly. Figure 1 provides schematic representations of the kinds of displays used in the experiment. The figure simply sets out the different types of colored shapes that were present in a given display: it does not convey the actual spatial layout of any actual display. The shapes were randomly positioned around a central fixation point in a non-overlapping fashion and the shapes’ positions were randomly determined prior to each trial.

The three critical conditions were as follows and were defined with respect to particular kinds of displays (see Figure 1). In the Shape_Diff/Col_Same displays, there were two different kinds of shapes present in the same color. In the Shape_Diff/Comp displays, two different kinds of colored shapes were present; hence, type of shape and type of color were perfectly correlated (these are the so-called ‘compatible’ cases). Finally, in the Shape_Diff/Incomp displays, there were two different kinds of shapes and two different kinds of colors, but shape distinction and the color distinction did not coincide (these were ‘incompatible’ cases).

Gestalt principles of grouping predict that performance will be most efficient when the type of color and the type of shape correlate perfectly. This is because grouping according to a common shape and grouping according to a common color coincide. It will be least efficient when common shape and, separately, common color suggest different groupings [11]. This is because it is assumed that the strength of a grouping is, in part, determined by the strength of similarity between the items that are being grouped [12]. Thus, Gestalt principles of grouping predict that participants will respond quickest in the Shape_Diff/Comp condition because the shape and color distinctions are coincident and grouping will be facilitated. In contrast, participants should respond slowest in the Shape_Diff/Incomp condition because the conflicting shape and color differences will inhibit grouping. We can think of the Shape_Diff/Col_Same condition as something of a control condition when the counting of different shapes within the same group takes place.

For completeness, we also included two control conditions. In the Shape_Same/Col_Same displays, all the shapes were the same and they all shared a common color. The Shape_Same/Col_Diff displays contained two differently colored tokens of the same type of shape. Any contrast in performance across these two conditions allowed us to examine the degree to which an irrelevant color difference across the shapes affected performance. Remember, in order to complete the task efficiently, participants had to discount any color differences that might have been present in the displays.

Deriving predictions of performance in our shape counting task from BM theory is not straightforward. The key processing constraint in BM theory is the number of maps that need to be constructed and consulted in order to complete the task. However, the picture is more nuanced than this. In keeping with feature integration theory [1], BM theory accepts that, fundamentally, perceptual information is registered on independent maps. Perception ultimately depends on the kinds of Boolean maps that are then created and consulted. A crucial distinction, however, is drawn between ‘access’ and ‘selection’ [2,3,4,5], such that, “Access defines the limit … of visual information that is able to reach the stage of consciousness at any one moment. Selection …governs what gains access to the stage of consciousness” [2] (p. 162). The basic idea is that items in a display can be easily selected if they share a salient common property. However, such selection alone may not support item identification. Merely selecting the green items from a display of red and green items is not sufficient itself to indicate which of the green items is a letter ‘T’, for example. In BM theory, the claim is that additional constraints apply once items have been selected on the basis of a particular common feature.

In particular, a Boolean map is the linkage of one feature value per dimension with a set of locations [2]: all the items coded on the RED map are red and from this map representation the items’ other perceptual characteristics can be accessed. In the case where the display contains only one red item, its shape is easy to access because the RED map also codes a single shape. However, accessing shape information is compromised when different types of red shapes are present. Different shapes are not explicitly represented on the same color map. Hence, accessing shape information must proceed on an item-by-item basis.

Given this, consider a display containing a red circle on the right and a green square on the left. Let us assume that (for whatever reason) a RED map is constructed: this map captures a red region on the left such that this region is also registered as a circle. This is because, “even if a Boolean map can simultaneously contain only one feature from each dimension it can simultaneously contain multiple feature labels from multiple different dimensions” [2] (p. 164), with the additional proviso that only one other type of feature label per dimension can be so coded. As long as the display contains only red circles then both ‘red’ and ‘circle’ are coded on the Boolean color map. Critically, though, if the green square were to be replaced by a red square, then the representation of the two shapes (i.e., ‘circle’ and ‘square’) is indeterminate on the RED map and hence shape information is now inaccessible.

Given this understanding of BM theory, we may now derive predictions for the current shape counting task. In BM theory it is claimed that the generation of Boolean maps is under conscious control [5]; therefore, the optimal strategy is to construct and consult Boolean maps for shape because the task demands judging the most prevalent shape. As a consequence, BM theory predicts that performance would be equivalent in all of the critical conditions. This is because, in all cases, two shape maps need to be consulted to determine the most prevalent shape. Since this prediction contrasts with that of the grouping account, the speeded counting task promises to provide a way of deciding between these competing hypotheses. We consider in the general discussion whether more complex predictions can be made from the BM theory by relaxing the strictures of the original theory.

Two experiments were carried out. The two experiments were identical except in one regard: in Experiment 1, there was no response deadline and the stimulus display remained on until a response was registered. In Experiment 2, participants had a response deadline of 1.5 s, and so participants were pressed to respond quickly. On every trial, the stimulus display comprised randomly positioned geometrical shapes (only squares, only circles, or a mixture of both—see Figure 1). The task was to decide as quickly and accurately as possible whether there were more squares or more circles.

## 2. Experiment 1

### 2.1. Participants

In Experiment 1, data from 30 participants were collected (mean age 21 years). All of the participants were second-year undergraduates who received course credit as remuneration.

### 2.2. Procedure and Design

The onset of each trial was signaled by the presentation of a central dot for 500 ms. Immediately following the stimulus, the display was presented and participants were instructed to decide whether there were more squares or more circles present. To respond ‘more squares’ they pressed the ‘K’ keyboard key and to respond ‘more circles’ they pressed the ‘D’ key. They were instructed to respond as quickly and accurately as possible. Visual feedback of either ‘Correct’ or ‘Error’ was presented for 300 ms, and following a blank of another 300 ms, the next trial started.

Each shape’s location was chosen at random within an unmarked rectangular area (width—280 pixels; height—360 pixels). Shapes were drawn as non-overlapping. The circle had a radius of 10 pixels and the square was defined to be equivalent in area to the circle. The background of the screen was black and the color of the shapes was determined on a quasi-random fashion from the following PC colors: red, green, blue and yellow. The assignment of the shapes and colors was determined according to the trial type.

There were five basic conditions replicated across three display set sizes (see Figure 1). Displays either contained three, five or seven shapes. Two control conditions were designated (i) Shape_Same/Col_Same: displays in which all the shapes were the same and they all shared a common color, or (ii) Shape_Same/Col_Diff: displays in which all the shapes were the same but the larger and smaller subsets were presented in different colors. In the critical conditions, the larger subset comprised one kind of shape (circles or squares) and the smaller subset comprised the other kind. The conditions were designated (i) Shape_Diff/Col_Same: all of the shapes shared a common color, (ii) Shape_Diff/Comp: displays in which type of color and type of shape were perfectly correlated (for instance, three green circles and two red squares—these were the compatible cases), or (iii) Shape_Diff/Incomp: displays in which type of color did not map perfectly onto the type of shape (for instance, two green circles, two red squares and one red circle—these were the incompatible cases).

The experimental script was written in JavaScript in the context of the JSPsych library [13]. Once a participant had agreed by email to complete the experiment, they were forwarded a link to the experimental script. As a consequence, each participant ran the experiment on whatever computer they had access to, and they were at liberty to test themselves wherever and whenever they wanted. They were asked to test themselves in a quiet place away from distractions and interruptions. Accessing the link launched the script that ran in their own web browser in full screen mode. There were 30 different kinds of trial type in total (15 each for the square and circle response, respectively) and initially participants ran through a random order of these in an initial block of practice trials. Next, six blocks of experimental trials were presented. Each block comprised 120 trials constituted by 4 random orders of the 30 trial types. At the end of each block a pause message was presented, and the participant self-initiated the next block of trials with a key response on the keyboard.

### 2.3. Results

#### 2.3.1. Control Conditions

**Figure 2:**
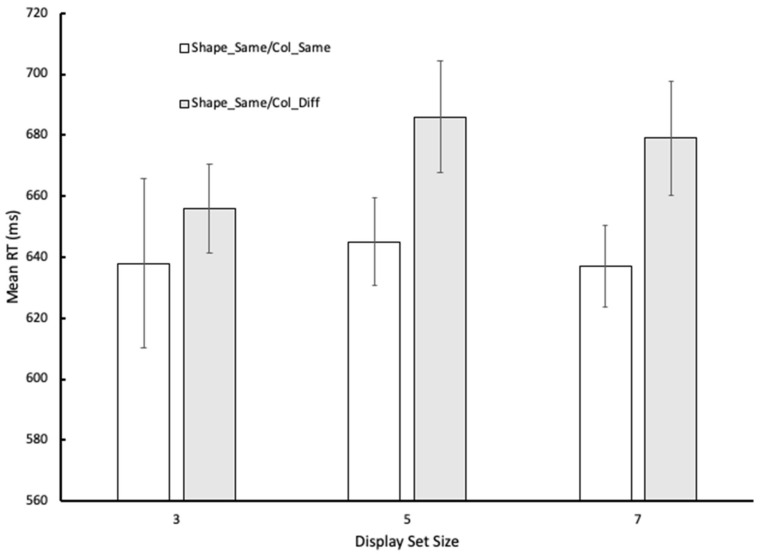
Graphical illustration of the mean RTs for the control conditions in Experiment 1. Error bars reflect 95% CIs as recommended by Bakeman and McArthur [14].

We began by focusing on performance in the two control conditions (see Figure 2 for a graphical summary of the mean RTs). The mean RTs per condition of interest for each participant were entered into a 3 × 2 repeated-measures ANOVA in which display set size (three, five, and seven) and condition (Shape_Same/Col_Same vs. Shape_Same/Col_Diff) acted as fixed factors and participants acted as a random factor. The analysis revealed that only the main effect of condition reached statistical significance: *F* (1, 29) = 21.85, *p* < 0.001, $\eta_{p}^{2}$ = 0.43; *F* (2, 58) = 1.04, *p* > 0.05, $\eta_{p}^{2}$ = 0.04, for the main effect of display set size; and *F* (2, 58) = 2.01, *p* > 0. 05, $\eta_{p}^{2}$ = 0.07, for the condition x display set size interaction. Corresponding error rates did not exceed 8% and when mean error rates were analyzed in a similar fashion to RTs none of the tests reached statistical significance at the 0.05 level. There was no evidence of any systematic speed/error trade-offs (see Table 1 for summaries of the measures of accuracy). In sum, for displays containing only a single shape type, participants were slower in their shape prevalence judgments when two colors were present than when all the shapes shared a common color.

#### 2.3.2. Critical Conditions

**Figure 3:**
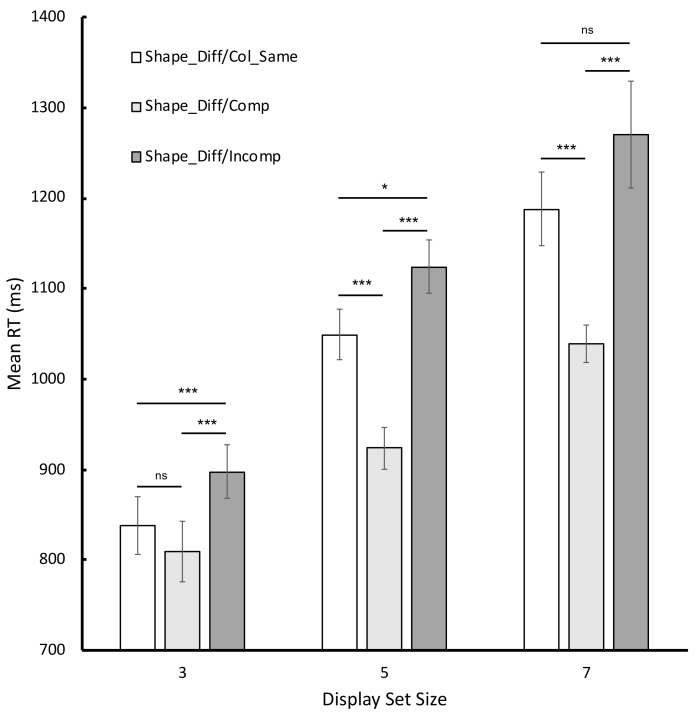
Graphical illustration of the mean RTs for the critical conditions in Experiment 1. Error bars reflect 95% CIs as recommended by Bakeman and McArthur [14]. *ns* signifies not statistically reliable at the 0.05 level; *** signifies *p* < 0.001; * signifies *p* < 0.05 (Bonferroni corrected).

Turning to the data from the critical conditions, the corresponding mean RTs were entered into a 3 × 3 repeated-measures ANOVA in which display set size (as before) and condition (Shape_Diff/Col_Same, Shape_Diff/Compatible and Shape_Diff/Incompatible) were entered as fixed factors and participants acted as a random factor (see Figure 3 for a graphical illustration of the corresponding RT data). The analysis revealed statistically significant main effects of both display set size, *F* (2, 58) = 130.03, *p* < 0.001, $\eta_{p}^{2}$ = 0.82, and condition, *F* (2, 58) = 67.69, *p* < 0.001, $\eta_{p}^{2}$ = 0.70, and a statistically significant display set size x condition interaction, *F* (4, 116) = 8.85, *p* < 0.001, $\eta_{p}^{2}$ = 0.23. Results of the Bonferroni-corrected simple main effects are shown in Figure 3. Most importantly, for all three display set sizes, participants were slower to respond to incompatible cases than compatible cases (and this difference scaled directly with display set size). This critical finding favors an account of processing based on grouping rather than one that posits constraints about the sequential interrogation of functionally separate feature maps.

A corresponding ANOVA on the error rates mirrored the pattern of significance reported for the RTs. The analysis revealed statistically significant main effects of both display set size, *F* (2, 58) = 29.56, *p* < 0.001, $\eta_{p}^{2}$ = 0.51, and condition, *F* (2, 58) = 33.83, *p* < 0.001, $\eta_{p}^{2}$ = 0.54, together with a statistically significant display set size x condition interaction, *F* (4, 116) = 9.18, *p* < 0.001, $\eta_{p}^{2}$ = 0.24. In all cases, participants were less accurate with incompatible displays than the compatible displays (although for display set size 3, this difference only approached statistical significance, *p* = 0.073, Bonferroni corrected). As with the RT data, the compatibility effect scaled directly with display set size. There was no evidence of any systematic speed/error trade-offs.

## 3. Experiment 2

### 3.1. Participants

In Experiment 2, 27 participants were recruited from the York Psychology Department’s participant panel. The panel predominantly comprised student members of the university. Remuneration was in terms of a £5 Amazon voucher or course credit. One participant’s data were omitted from consideration due to a high prevalence of missed responses (72% of trials were either missed trials or errors). The mean age of the remaining 26 participants was 22 years.

### 3.2. Procedure

The only difference between Experiment 1 and 2 was the presence of a 1.5 s response deadline. In Experiment 2, if the computer failed to detect a keyboard response within 1.5 s it moved onto the next trial. Participants in Experiment 2 were also instructed at the start of testing about the 1.5 s time limit on responding.

### 3.3. Results

#### 3.3.1. Control Conditions

**Figure 4:**
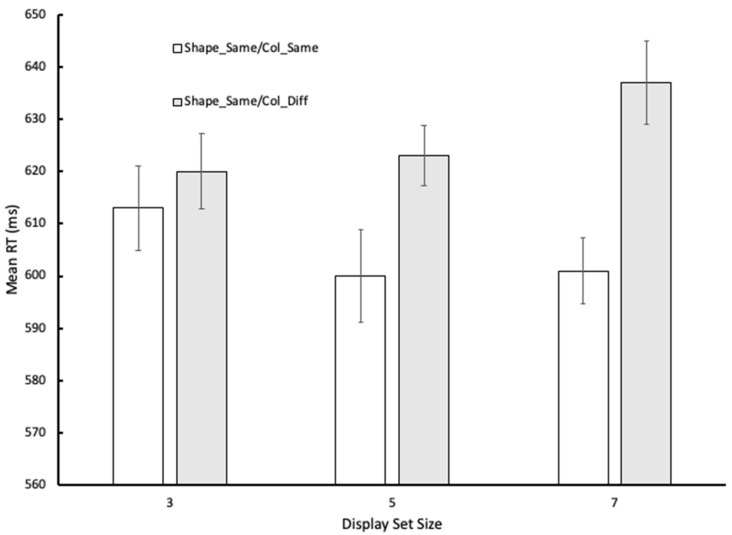
Graphical illustration of the mean RTs for the control conditions in Experiment 2. Error bars reflect 95% Cis, as recommended by Bakeman and McArthur [14].

The analysis of the RT data revealed that both the main effect of condition, *F* (1, 25) = 45.25, *p* < 0.001, $\eta_{p}^{2}$ = 0.64, and the condition x display set size interaction, *F* (2, 50) = 11.94, *p* < 0.001, $\eta_{p}^{2}$ = 0.32, reached statistical significance, *F* (2, 50) = 1.30, *p* > 0.05, $\eta_{p}^{2}$ = 0.05, for the main effect of display set size. The results in Experiment 2 largely mirror the effects reported for Experiment 1—the only slight discrepancy is that whereas, in this case, the condition x display set size interaction was statistically reliable, it failed to reach statistical significance in the data for Experiment 1 (there *p* = 0.06). Nonetheless, the pattern of responding was the same, with the effect of condition being less marked with display set size 3 than with the larger displays. A graphical illustration of the RT data can be found in Figure 4.

Accuracy levels were generally high with no error rate exceeding 6% in any condition (see Table 2). Analysis revealed that both the main effect of condition, *F* (1, 25) = 4.88, *p* < 0.05, $\eta_{p}^{2}$ = 0.16, and the condition x display set size interaction, *F* (2, 50) = 3.33, *p* < 0.05, $\eta_{p}^{2}$ = 0.12, reached statistical significance, *F* < 1.0 for the main effect of display set size. These effects were particularly small and appeared to be carried by the pattern of responding for display set size 5. There is no apparent reason for this.

#### 3.3.2. Critical Conditions

**Figure 5:**
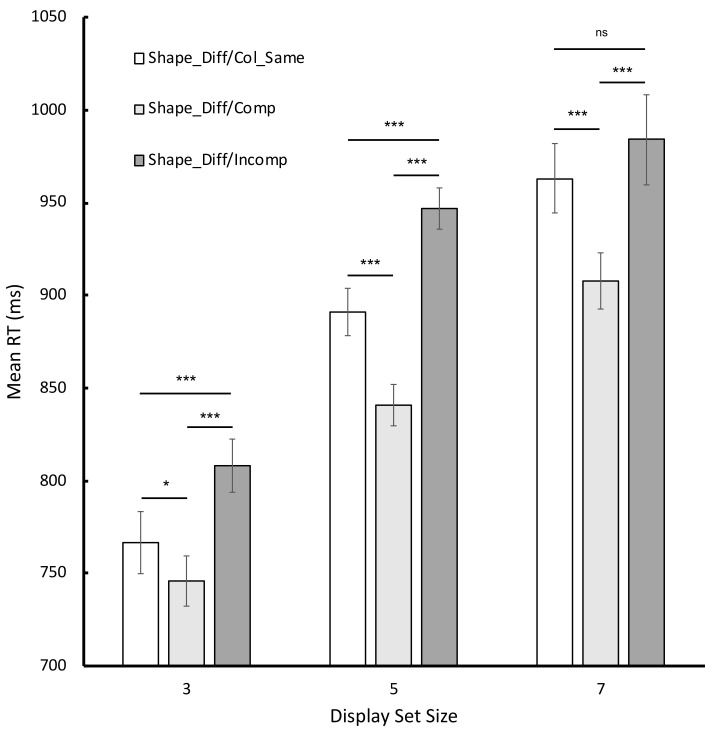
Graphical illustration of the mean RTs for the critical conditions in Experiment 2. Error bars reflect 95% CIs as recommended by Bakeman and McArthur [14]. *ns* signifies not statistically reliable at the 0.05 level; *** signifies *p* <0.001; * signifies *p* < 0.05 (Bonferroni corrected).

The analysis revealed statistically significant main effects of both display set size, *F* (2, 50) = 182.50, *p* < 0.001, $\eta_{p}^{2}$ = 0.88, and condition, *F* (2, 50) = 119.74, *p* < 0.001, $\eta_{p}^{2}$ = 0.83, and a statistically significant display set size x condition interaction, *F* (4, 110) = 4.34, *p* < 0.01, $\eta_{p}^{2}$ = 0.15. The results of the Bonferroni-corrected simple main effects are shown in Figure 5. Critically for all three display set sizes, participants were slower to respond to incompatible cases than compatible cases, supporting the grouping theory over BM theory. A corresponding ANOVA on the error rates mirrored the pattern of significance reported for the RTs. There was no evidence of any systematic speed/error trade-offs. A graphical illustration of the RT data can be found in Figure 5.

## 4. Discussion

The results of both experiments were in general agreement, and are relatively clear cut. Participants were faster in making shape prevalence judgments when the type of shape and type of color were perfectly correlated (in the compatible displays) than when one instance of one kind of shape shared its color with the tokens of the other shape type (in the incompatible displays). This pattern of performance was predicted by the theory of processing based on Gestalt principles of grouping in which the strength of grouping is, in part, determined by the strength of similarity of the items that are being grouped. Importantly, grouping is seen to be an interactive and competitive process in which information derived from concurrently presented perceptual dimensions determines the perceptual organization of the input [9,10,11]. The present findings sit less well with BM theory which posits constraints that operate as a consequence of the derivation of featural information from a sequence of Boolean shape maps.

More specifically, the data are readily explicable within the framework sketched out by Quinlan and Wilton [11], who argue that early grouping processes operate to divide the individual display elements into clusters that may define an object or object part. The evidence they report is indicative of grouping primarily on the basis of proximity. According to Quinlan and Wilton [11], once such clusters have been derived, additional processes are then invoked to establish whether the elements within a cluster are of the same type. Here, in the absence of any systematic manipulation of proximity, the evidence reveals grouping on the basis of the alignment of features from across perceptual dimensions, in line with the writings of Kubovy and colleagues [9,10]. A theory of processing based on Gestalt principles of grouping thereby provides a parsimonious account of the current results.

In contrast, the data do not accord well with the basic tenets of BM theory. According to the most straightforward interpretation of the theory, BM theory predicts that performance would be equivalent in all of the critical conditions, because in all those cases, two shape maps need to be consulted to determine the most prevalent shape. This, however, is not what we found.

We are mindful that because BM theory is not well-specified, it is possible to conjure ad hoc explanations for the data that appear to follow from the theory. We therefore consider how such a version of the theory might work. First, contrary to the claims of Huang and Pashler [5] that the generation of Boolean maps is under conscious control, it might be asserted that in special cases performance may be driven by the automatic construction of color Boolean maps. For example, in the compatible condition, the salient color difference in the displays will drive the creation of two separate color maps. Within each of these maps, a common shape is encoded and shape counting proceeds with respect to these two maps. In the incompatible condition, the color difference again drives the creation of two-color maps; in one of the maps, a common shape is coded but the other contains different shape types, and hence accessing the items’ shapes is compromised. In the Shape_Diff/Col_Same condition, where there is no salient color difference, counting takes place on two separate shape maps.

This interpretation of BM theory provides a very clear set of predictions. Shape counting for the Shape_Diff/Col_Same and the compatible cases proceeds via shape access on two different feature maps—two-shape maps and two-color maps, respectively—hence RT should be equivalent across these two conditions. However, because shape access is compromised in the incompatible case, there will be a RT penalty relative to the other two conditions. As this is not the pattern that we report, we conclude that simply accepting that color differences will automatically produce color Boolean maps fails to predict the overall speeding in the compatible condition and therefore fails to accommodate the pattern of performance found in our experiments. Indeed, we believe such an assumption is counter to BM theory because of the claim that the creation of Boolean maps is under conscious control. The nature of the colors of the shapes is completely irrelevant to the successful completion of the task. Participants should, therefore, consciously strive to construct shape and not color maps.

To account for our data within a BM framework, it must be assumed that the system always produces Boolean maps based on color first—even in the absence of any color difference. By this assumption, a color Boolean map is constructed even in the Shape_Diff/Col_Same case. Performance in the compatible condition will be fast because two color maps are created and a single type of shape is registered on each map. Shape counting can then proceed relative to these two maps. Performance in Shape_Diff/Col_Same case will be relatively slower because shape information is inaccessible from the single-color map that is created, and so two additional shape maps will need to be constructed and consulted. Finally, performance in the incompatible condition will be slowest overall, because on one of the color maps that is created shape information is inaccessible. On these grounds, alternative shape maps must be constructed and consulted.

In discussing such a BM account, we accept that the data can be accommodated if a key ad hoc assumption about the generation of color maps is accepted. This assumption rests on additional processing constraints that are not present in the original formulation of the theory and do not appear to be justified on grounds other than that they are needed to account for the data. As Popper [15] stated, “we can always immunize a theory against refutation” (p. 357), but in doing so, the theory becomes unscientific because it is impossible to know the conditions under which it could be falsified. In contrast, the alternative theory of grouping naturally accounts for the data.

Further support for this conclusion comes from complementary evidence that supports an account based on grouping principles, and contradicts the predictions of BM theory. In an experiment very similar to Experiment 2 described by Huang [2], Müller and O’Grady [16] presented participants with a brief masked display containing two overlapping rectangles: a vertically aligned and a horizontally aligned case, respectively. Each rectangle was associated with two dimensions, namely, shape and color, and within each dimension two values were tested. In the shape dimension, the rectangle could be either large or small (two different size values) and its boundary could either be dashed or solid (two different texture values). With respect to the color dimension, the rectangle could be either be red or yellow (two different hue values), and its saturation was either high or low. In the within-dimension conditions, participants reported on the values from the color (hue and saturation) or the shape dimension (size and texture); in the across-dimension condition they reported on one of the possible shape values (either size or texture) and one of the color values (either hue or saturation). In the single-object cases, participants were instructed to report only attributes of one of the rectangles (either horizontal or vertical), and in the dual-object cases they were instructed to report on attributes of both rectangles.

In their across-dimensions conditions, Müller and O’Grady [16] reported that performance was significantly worse when participants were instructed to divide attention across two objects than when they were instructed to focus attention on a single object, and this is the exact pattern that was found by Huang [2]. We shall refer to this finding as a ‘dual-object cost’ and Huang’s findings simply replicate and extend this dual-object cost. However, data reported by Müller and O’Grady [16] also reveal a ‘dual-dimension cost’. That is, participants were more accurate in their responses when they were asked to report values from the same dimensions (color: hue and saturation, or shape: size and texture) than they were when they were asked to divide attention across dimensions. Moreover, this dual-dimension cost occurred when participants were asked to report attributes from a single object. Overall, the data revealed a dual-object cost and a dual-dimension cost and these costs were additive with one another. In this regard, there are effects of both dimensions and of objects and they are not interchangeable, contra Huang [2] (p. 175).

Critically, the dual-dimension costs are not readily predicted by BM theory. On the understanding that such a Boolean map codes unique values on multiple dimensions, then there is nothing in the theory to explain why different features of the same dimension are more accurately reported than features of different dimensions. Of course, this constraint might be added to the theoretical account in the same way that the theory can be extended to explain our own data, but other alternatives are perhaps more attractive. For example, in closing their discussion, Müller and O’Grady set out an account of their data that sits well with the ideas of perceptual organization discussed here. Put simply, “Domain-based selection, considered to be a form of segmentation, is assumed to occur first followed by object-based selection... e.g., segmentation driven by a weighted domain, d_1_, allows one object, o_1_, to be passed on for further processing making the domain-specific attributes of o_1_ available for report” [16] (p. 1349). The patterns of performance observed are then readily explained in terms of the dimensions to be reported, given which objects are to be considered.

## 5. Conclusions

In summary, here we have described two new experiments that illuminate how objects and their properties are processed in vision. The overall aim was to pit traditional ideas about principles of perceptual organization with more recent ideas encapsulated in BM theory. We have argued that the present findings fit more comfortably with notions of perceptual grouping than with BM theory. Our data provide further support for the claim that the grouping of elements into plausible figures against a plausible background is a key property of early vision. Such figure/ground segmentation is based on an early analysis of the perceptual dimensions that present in the optic array. In line with Müller and O’Grady [16], we assert that the most fruitful way to understand the operation of the human visual system is to assume that dimensional processing is a critical precursor to the derivation of visual objects. The manner in which attention can then be deployed is, in turn, critically dependent on both perceptual objects and their properties.

## Figures and Tables

**Table 1:** Average error rates (expressed as proportions) for the conditions of interest in Experiment 1.

Display Type	Display Set Size		
	3	5	7
Shape_Diff/Col_Same	0.12	0.13	0.18
Shape_Diff/Comp	0.10	0.11	0.14
Shape_Diff/Incomp	0.12	0.14	0.24
Shape_Same/Col_Same	0.07	0.05	0.06
Shape_Same/Col_Diff	0.08	0.06	0.06

**Table 2:** Average error and miss rates (expressed as proportions) for the conditions of interest in Experiment 2.

Display Type	Display Set Size					
	3		5		7	
	Err	Miss	Err	Miss	Err	Miss
Shape_Diff/Col_Same	0.11	0.01	0.13	0.02	0.21	0.05
Shape_Diff/Comp	0.08	0.01	0.10	0.01	0.15	0.02
Shape_Diff/Incomp	0.13	0.01	0.17	0.03	0.27	0.07
Shape_Same/Col_Same	0.04	0.00	0.05	0.00	0.04	0.00
Shape_Same/Col_Diff	0.04	0.00	0.03	0.00	0.04	0.00

## Data Availability

Raw data files are accessible at https://pure.york.ac.uk/portal/en/datasets/bm-data-sets(11d628ed-f47e-4d24-bb03-8faf349eda50).html (accessed on 15 August 2022). The first author can provide access to the experimental scripts. The experiments were not preregistered.
